# Curcumin protects against bisphenol A‐induced hepatic steatosis by inhibiting cholesterol absorption and synthesis in CD‐1 mice

**DOI:** 10.1002/fsn3.3468

**Published:** 2023-06-01

**Authors:** Ting Hong, Jun Zou, Jie Yang, Hao Liu, Zhuo Cao, Youming He, Dan Feng

**Affiliations:** ^1^ Guangdong Provincial Key Laboratory of Food, Nutrition and Health, Department of Nutrition, School of Public Health Sun Yat‐sen University Guangzhou China; ^2^ Guangzhou Key Laboratory of Environmental Pollution and Health Risk Assessment, Department of Occupational and Environmental Health, School of Public Health Sun Yat‐sen University Guangzhou China; ^3^ Department of Cardiology The Sixth Affiliated Hospital of South China University of Technology Foshan China

**Keywords:** 3‐hydroxy‐3‐methylglutaryl coenzyme A reductase, bisphenol A, curcumin, hepatic steatosis, Niemann–Pick C1‐like 1

## Abstract

Curcumin is a polyphenol extracted from the rhizome of turmeric, and our previous research showed that curcumin inhibited cholesterol absorption and had cholesterol‐lowering effect. Bisphenol A (BPA), a common plasticizer, is widely used in the manufacture of food packaging and is associated with non‐alcoholic fatty liver disease (NAFLD). We hypothesized that curcumin could protect against BPA‐induced hepatic steatosis by inhibiting cholesterol absorption and synthesis. Male CD‐1 mice fed BPA‐contaminated diet with or without curcumin for 24 weeks were used to test our hypothesis. We found that chronic low‐dose BPA exposure significantly increased the levels of serum triglyceride (TG), total cholesterol (TC), and low‐density lipoprotein cholesterol and the contents of liver TG and TC, resulting in liver fat accumulation and hepatic steatosis while curcumin supplementation could alleviate BPA‐induced dyslipidemia and hepatic steatosis. Moreover, the anti‐steatosis and cholesterol‐lowering effects of curcumin against BPA coincided with a significant reduction in intestinal cholesterol absorption and liver cholesterol synthesis, which was modulated by suppressing the expression of sterol regulatory element‐binding protein‐2 (SREBP‐2), Niemann–Pick C1‐like 1 (NPC1L1), and 3‐hydroxy‐3‐methylglutaryl coenzyme A reductase (HMGCR) in the small intestine and liver. In addition, the expression levels of liver lipogenic genes such as liver X receptor alpha (LXRα), SREBP‐1c, acetyl‐CoA carboxylase 1 (ACC1), and ACC2 were also markedly down‐regulated by curcumin. Overall, our findings indicated that curcumin inhibited BPA‐induced intestinal cholesterol absorption and liver cholesterol synthesis by suppressing SREBP‐2, NPC1L1, and HMGCR expression, subsequently reducing liver cholesterol accumulation and fat synthesis, thereby preventing hepatic steatosis and NAFLD.

## Graphical abstract

Curcumin inhibited bisphenol A‐induced intestinal cholesterol absorption and liver cholesterol synthesis by suppressing sterol regulatory element‐binding protein‐2, Niemann–Pick C1‐like 1, and 3‐hydroxy‐3‐methylglutaryl coenzyme A reductase expression, thus ameliorating hepatic steatosis induced by bisphenol A in male CD‐1 mice.

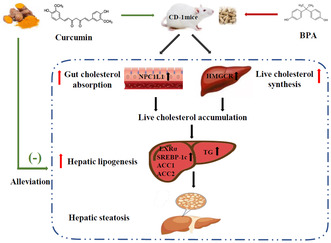

## 1 INTRODUCTION

Non‐alcoholic fatty liver disease (NAFLD) is a worldwide prevalent metabolic liver disease and causes heavy disease burden (Paik et al., 2021). The earliest and core feature of NAFLD is hepatic steatosis, which is characterized by liver fat accumulation (Starekova et al., 2021). As the prevalence of NAFLD soars worldwide in recent years, it is urgent to improve the medical and dietary strategies to prevent NAFLD or reverse the pathological progression in the early phase.

The underlying mechanisms of NAFLD are complicated and have not yet been fully elucidated (Teng et al., 2022). Recently, the disorder of cholesterol metabolism has been reported to be closely associated with NAFLD (Malhotra et al., 2020). Intestinal cholesterol absorption and hepatic cholesterol synthesis have crucial roles in maintaining cholesterol homeostasis (Luo et al., 2020). Excessive intestinal cholesterol absorption and hepatic cholesterol synthesis can elevate serum cholesterol levels and increase liver cholesterol accumulation, which then stimulates liver fat synthesis and promotes liver lipid accumulation, leading to hepatic steatosis and NAFLD (Arguello et al., 2015). Liver lipid synthesis is mainly modulated by sterol regulatory element‐binding proteins (SREBPs) and metabolic enzymes (Park et al., 2018). Increased hepatic cholesterol accumulation can activate hepatic liver X receptor alpha (LXRα) and enhance SREBP‐1c expression, which, in turn, activates the metabolic enzyme acetyl‐CoA carboxylase 1 (ACC1) and ACC2, resulting in increased hepatic lipid synthesis and lipid accumulation (Arguello et al., 2015; Moslehi & Hamidi‐Zad, 2018).

NAFLD is affected by many factors, including environmental and dietary factors. Bisphenol A (BPA), a common plasticizer with an endocrine‐disrupting effect, is widely used in the manufacture of food packaging and storage containers due to its excellent physical and chemical properties. The primary exposure pathway of BPA in humans is oral intake because BPA can migrate from containers to food and beverages (Tarafdar et al., 2022). Although the tolerable daily intake (TDI) of BPA is 50 μg/kg/day, several animal and human studies have indicated that exposure to low‐dose BPA disrupts multiple biological processes, for example, reproduction, development, and metabolism (Martínez‐Ibarra et al., 2021). Epidemiological investigations revealed that long‐term exposure to low‐dose BPA increased NAFLD risk (Verstraete et al., 2018). Serum and urine BPA levels in NAFLD patients were higher than those in healthy people (Dallio et al., 2018; Kim et al., 2019). Our previous experiments showed that exposure to 50 μg/kg/day BPA induced liver cholesterol synthesis and significant hepatic steatosis in mice (Feng et al., 2020; Li, Zhang, Zou, Mai, et al., 2019). In Caco‐2 cells, we also found that exposure to environmentally relevant dose of BPA induced the expression of Niemann‐Pick C1‐like 1 (NPC1L1) and cellular cholesterol absorption (Feng, Zou, Zhang, Li, Li, & Lu, 2017).

Existing evidence has shown that some plant‐active substances in the diet can effectively lower cholesterol levels and improve hepatic steatosis (Feng et al., 2019; Feng, Zou, Zhang, Li, & Lu, 2017; Khan et al., 2019; Srinivasan, 2013). Curcumin is a polyphenol phytochemical substance extracted from the rhizome of turmeric and is very common in daily diet, such as mustard and curry (Sharifi‐Rad et al., 2020). After years of research and exploration, this polyphenol has been proved to possess anti‐inflammatory, antioxidant, hypoglycemic, and liver‐protective properties (Feng et al., 2019; Feng, Zou, Zhang, Li, & Lu, 2017; Zhang et al., 2018), and has the potential to treat diabetes, obesity, NAFLD, and other diseases (Khan et al., 2019; Różański et al., 2021; Thota et al., 2020). In addition, our and other research demonstrated that curcumin regulated cholesterol absorption and synthesis and possessed cholesterol‐lowering effects (Feng et al., 2010; Feng, Zou, Zhang, Li, & Lu, 2017; Liu et al., 2017). Recently, curcumin has also been reported to prevent the damage caused by BPA (Facina et al., 2021; Geng et al., 2017; Tandon et al., 2020). However, whether curcumin can protect against BPA‐induced hepatic steatosis through modulating cholesterol absorption and synthesis and liver fat biosynthesis remains to be elucidated.

According to existing research, we hypothesized that curcumin can suppress BPA‐induced intestinal cholesterol absorption and hepatic cholesterol synthesis, then reduce serum and liver cholesterol concentration, thereby alleviating liver fat synthesis and preventing hepatic steatosis. In this study, male CD‐1 mice fed BPA‐contaminated diet with or without curcumin for 24 weeks were used to test our hypothesis. Enzymological methods and liver tissue staining were used to determine hepatic lipid homeostasis. RT‐qPCR and western blotting were further performed to analyze the expressions of genes responsible for intestinal cholesterol absorption and hepatic lipid synthesis. Our study would contribute to the application of curcumin as a potential and new strategy for the prevention and therapy of BPA‐induced hepatic steatosis in the future.

## 2 METHODS AND MATERIALS

### 2.1 Animals and diets

Twenty‐four male CD‐1 mice at 5 weeks of age were purchased from Beijing Vital River Laboratory Animal Technology Co., Ltd. The mice were raised in polypropylene cages with polypropylene water bottles and fed a normal diet for 1 week, and then randomly distributed into three groups (*n* = 8) according to the following diets: control group (MD12062 normal diet), BPA group (0.5 mg/kg BPA was incorporated in normal diet), and BPA + curcumin group (0.5 mg/kg BPA and 0.1% w/w were added to normal diet). Curcumin and BPA were obtained from Sigma‐Aldrich (purity ≥98%; St. Louis, MO, USA). The dose of 0.1% w/w curcumin has been reported to effectively lower cholesterol levels and improve hepatic steatosis (Hasan et al., 2014). The ingredient of the experimental diets was presented in Table 1. The daily diet of mice accounted for 10% of their body weight (Marmugi et al., 2012), so the actual daily intake of BPA is 50 μg/kg/day. The body weight and food intake of mice were monitored every week. Three days before the end of the experiment, the fecal materials of mice were taken and frozen at −80°C for further analysis. After fasting for 12 h, the mice were anesthetized and euthanized. The blood of orbital vein was gathered and centrifuged to collect serum. Liver and small intestine tissues were collected, weighed, and fixed for the following experiments, and the remaining tissues were frozen at −80°C.

**TABLE 1:** Ingredient composition of the experimental diets fed to CD‐1 mice.

Ingredient	Content per kilogram of feed
Energy, Kcal	4000
Fat, Kcal	158
Protein, Kcal	203
Carbohydrate, Kcal	640
Casein, g	200
Corn starch, g	397
Maltodextrin, g	132
Sucrose, g	100
Cellulose, g	50
Soya oil, g	70
Water, g	90

*Note*: The name of the diet and source, manufacturer: Diet MD 12062; Medicience Ltd, China.

All procedures were approved by the Institutional Animal Care and Use Committee of Sun Yat‐sen University (No. 2017–005) and were conducted following the National Research Council Guide for Care and Use of Laboratory Animals.

All reagents and chemicals involved in the test are provided in Table S1.

### 2.2 Biochemical analysis

The activity of serum alanine aminotransferase (ALT) and aspartate aminotransferase (AST) and the levels of serum total triglycerides (TG), total cholesterol (TC), low‐density lipoprotein cholesterol (LDL‐C), and high‐density lipoprotein cholesterol (HDL‐C) were determined by different assay kits (Jiancheng, Nanjing, China), respectively.

Hepatic TG and TC contents were detected as previously described (Feng et al., 2020). Briefly, the frozen liver tissue (50 mg) was homogenized in 1 mL lysate at 4°C, and then let it stand for 10 mi at 25°C. After heating at 70°C for 10 min and centrifuging at 400 *g* for 5 min, the supernatant was gathered and analyzed for liver TG and TC concentration by different assay kits, respectively. The liver TG and TC content was expressed as nmol of TG or TC per mg of protein.

### 2.3 Histological examination

The liver tissue in the middle part of left lobe was excised, fixed in 4% paraformaldehyde for 24 h at room temperature, paraffin embedded, cut into 5‐μm‐thick sections, and stained with hematoxylin–eosin (H&E) using standard techniques. Ten micrometer thickness of frozen sections from formalin‐fixed liver tissue were stained with Oil Red O. The image of Oil Red O staining was captured by optical microscope at 200 × magnification.

### 2.4 Real‐time quantitative polymerase chain reaction (RT‐qPCR) assay

Total RNA from liver tissue was isolated with TRIzol reagent and reverse transcribed into cDNA using Takara Prime Script™ RT reagent Kit. RT‐qPCR was performed with Applied Biosystems 7500 using the SYBR Green PCR master mix kit. The primers were synthesized by Sangon Biotech. The expression level of each gene was analyzed by 2^−ΔΔCt^ method and normalized with β‐actin. Primer sequences were presented in Table 2. Details were presented in Supplemental Method.

**TABLE 2:** Primers used in quantitative real‐time PCR.

Gene name	Primer sequences 5′ to 3′	GenBank accession
LXRα F	GGAGTGTCGACTTCGCAAATG	NM_013839
LXRα R	TCAAGCGGATCTGTTCTTCTGAC	
SREBP‐1c F	CAGACACTGGCCGAGATGTG	NM_011480
SREBP‐1c R	CTTGGTTGTTGATGAGCTGGAG	
ACC1 F	TTACAGGATGGTTTGGCCTTTC	NM_133360
ACC1 R	CAAATTCTGCTGGAGAAGCCAC	
ACC2 F	CCTGAATCTCACGCGCCTA	NM_133904
ACC2 R	CAGATGGAGTCCAGACATGCTG	
β‐Actin F	GCGTGACATCAAAGAGAAGC	NM_007393
β‐Actin R	CTCGTTGCCAATAGTGATGAC	

### 2.5 Western blotting assay

Western blotting was performed as previously described (Feng et al., 2020). In brief, total protein lysates of liver and small intestine were separated by 10% SDS‐PAGE and then transferred onto PVDF membrane. Firstly, primary polyclonal antibodies (anti‐SREBP‐2, anti‐HMGCR, anti‐NPC1L1, anti‐SREBP‐1c, anti‐LXRα, anti‐ACC1, anti‐ACC2, and anti‐β‐actin) were diluted (1:1000) and used to incubate with the membranes at 4°C for 12 h. Next, the membranes were incubated with secondary antibody (goat anti‐rabbit IgG‐HRP) at room temperature for 2 h. The bands were identified using chemiluminescence detection system, and the band intensities were measured using Image J software with β‐actin as an internal loading control. Details were presented in Supplemental Method.

### 2.6 Statistical analysis

Results were assessed by one‐way ANOVA or non‐parametric test. SNK‐*q* test or Kruskal–Wallis non‐parametric test was further adopted for pairwise comparisons among three groups. Data were analyzed using the SPSS package 21.0 and expressed as mean ± SEM. The graphs were produced by Priam 8.0 software. *p* < .05 was accepted as statistically significant.

## 3 RESULTS

### 3.1 Basic indices of CD‐1 mice

The liver weight and liver‐to‐body weight ratio were increased in BPA‐exposed mice compared to control mice after 24 weeks of dietary BPA exposure (*p* < .05). Compared to BPA‐exposed mice, mice in BPA + curcumin group exhibited lower liver weight and liver‐to‐body weight ratio, but there was no significant difference between the two groups of mice. In addition, the daily food intake and body weight were not significantly different among the three groups of mice (Table 3).

**TABLE 3:** Basic and liver parameters measured for CD‐1 mice.

	Group		
	Control	BPA	BPA + curcumin
Daily food intake(g)	4.41 ± 0.46	4.41 ± 0.21	4.39 ± 0.51
Body weight (g)	39.9 ± 2.79	41.5 ± 7.56	39.1 ± 3.80
Liver weight (g)	1.44 ± 0.10	1.79 ± 0.11*	1.65 ± 0.23
Liver/body weight (g/g)	0.03 ± 0.00	0.05 ± 0.00*	0.04 ± 0.00

*Note*: Results are mean ± SEM, *n* = 8. *P < .05 compared to control group.

Abbreviation: BPA, bisphenol A.

### 3.2 Curcumin improved blood biochemical changes induced by BPA

**FIGURE 1:**
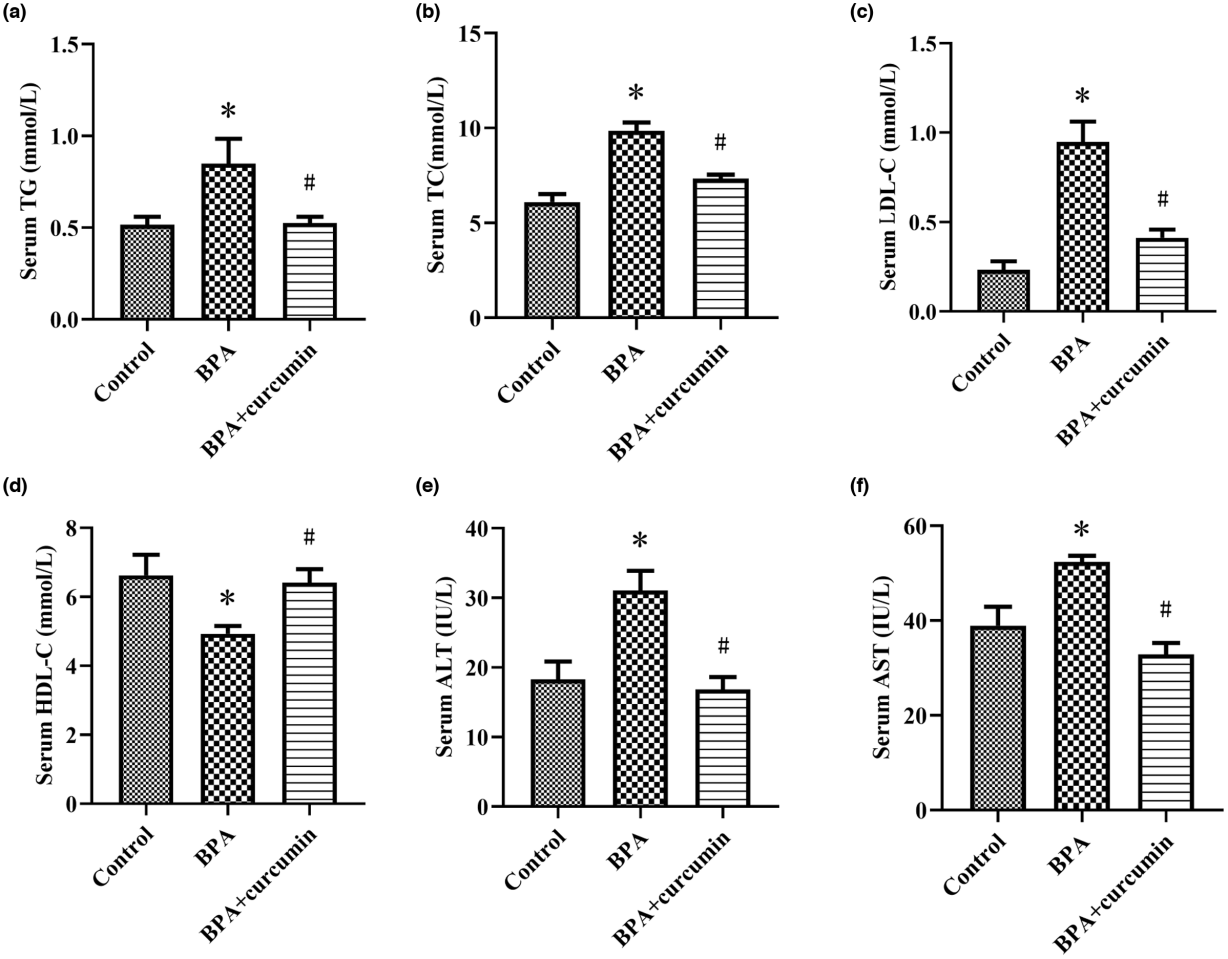
Bar plot of serum biochemical markers in CD‐1 mice. Male CD‐1 mice were fed BPA‐contaminated diet with or without curcumin for 24 weeks. (a–d) Serum TG, TC, LDL‐C, and HDL‐C levels; (e, f) Serum ALT and AST activity. Results are expressed as mean ± SEM (*n* = 8), *p < .05 versus control group, and ^#^ *p* < .05 versus BPA group.

In BPA‐exposed mice, serum TG, TC, and LDL‐C levels were noticeably increased and serum HDL‐C levels were reduced compared with control mice (*p* < .05). Elevated activities of serum AST and ALT were observed in BPA‐exposed mice (*p* < .05). It is noteworthy that these blood biochemical changes were significantly reversed by curcumin supplementation (Figure 1a–f, *p* < .05).

### 3.3 Curcumin improved liver histological changes and hepatic steatosis induced by BPA

**FIGURE 2:**
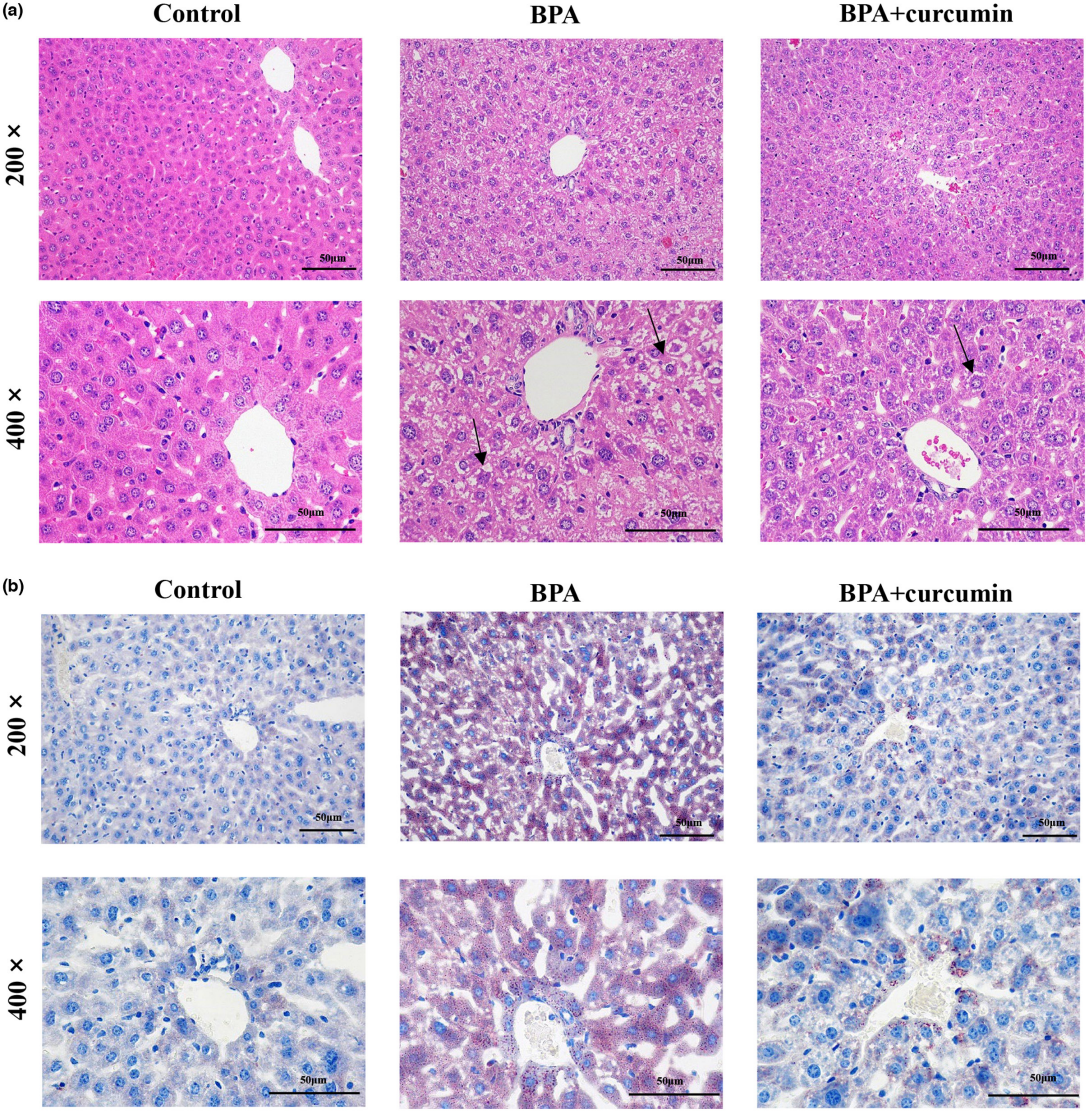
Curcumin improved liver histological changes and hepatic steatosis induced by BPA in CD‐1 mice. Male CD‐1 mice were fed BPA‐contaminated diet with or without curcumin for 24 weeks. (a) Representative pictures of H&E staining showing the liver morphological changes (200 **×** magnifications, 400 **×** magnifications); (b) Representative pictures of Oil Red O staining showing the liver fat accumulation (200 **×** magnifications, 400 **×** magnifications). The arrows refer to hepatocytes with significant histopathological changes.

H&E staining showed that the hepatocytes of control mice were normal and tightly arranged. On the contrary, the hepatocytes of BPA‐exposed mice exhibited disordered hepatic plate arrangement and abnormal morphology with many small vacuoles. While the liver histological changes caused by BPA were effectively reversed by curcumin, the liver cells tended to be normal in curcumin‐treated mice (Figure 2a).

Compared with control mice, Oil red O staining showed that massive fat droplets were accumulated in the hepatocytes of BPA‐exposed mice. However, curcumin intervention significantly reduced the accumulation of fat droplets caused by BPA (Figure 2b, Table 4, *p* < .05). Lipid quantitative analysis further demonstrated that liver TG and TC content in curcumin‐treated mice were also significantly reduced (Table 4, *p* < .05).

**TABLE 4:** Liver biochemical parameters measured for CD‐1 mice.

	Group		
	Control	BPA	BPA + curcumin
Liver TC (nmol/mg)	47.3 ± 1.78	53.9 ± 0.70*	38.5 ± 1.68^#^
Liver TG (nmol/mg)	18.9 ± 1.71	68.1 ± 10.8*	63.7 ± 4.57^#^
Lipid drops area (%)	0.77 ± 0.11	30.1 ± 4.67*	2.42 ± 0.23^#^

*Note*: Results are mean ± SEM, *n* = 8. *p < .05 compared to control group, ^#^ *p* < .05 compared to BPA group.

Abbreviations: BPA, bisphenol A; TC, total cholesterol; TG, total triglycerides.

### 3.4 Curcumin suppressed cholesterol synthesis and absorption induced by BPA

**FIGURE 3:**
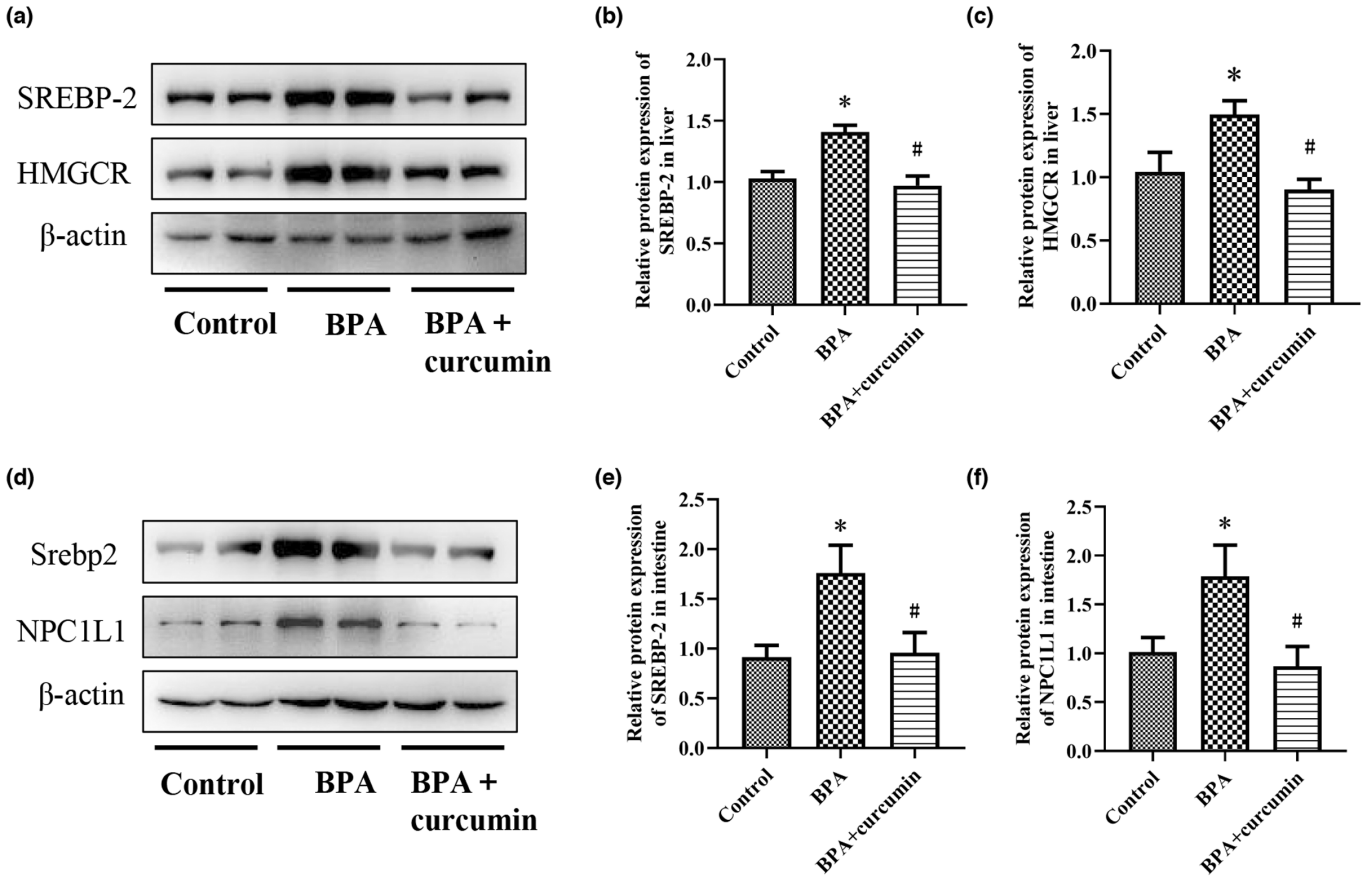
Curcumin inhibited the expression of SREBP‐2, HMGCR, and NPC1L1 induced by BPA in CD‐1 mice. Male CD‐1 mice were fed BPA‐contaminated diet with or without curcumin for 24 weeks. (a) The protein expression levels of SREBP‐2 and HMGCR in the liver; (b, c) The quantitative analysis of Western blotting assay for SREBP‐2 and HMGCR expression. (d) The protein expression levels of SREBP‐2 and NPC1L1 in the small intestine; (e, f) The quantitative analysis of Western blotting assay for SREBP‐2 and NPC1L1 expression. Results are expressed as mean ± SEM (*n* = 8), *p < .05 versus control group, and ^#^ *p* < .05 versus BPA group.

Excessive synthesis and absorption of cholesterol are predisposed to hepatic steatosis. In order to understand the mechanism by which BPA stimulated hepatic steatosis and the preventive effect of curcumin, we further measured the expression levels of cholesterol synthesis and absorption‐related proteins by western blotting experiments. For the cholesterol synthesis, liver SREBP‐2 and hepatic 3‐hydroxy‐3‐methylglutaryl coenzyme A reductase (HMGCR) were assayed. It was seen that BPA exposure significantly up‐regulated the expression of SREBP‐2 and HMGCR in the liver (*p* < .05), while curcumin intervention significantly decreased hepatic SREBP‐2 and HMGCR expression (Figure 3a–c, *p* < .05). For the cholesterol absorption, intestinal SREBP‐2 and NPC1L1 were selected for measurement. Notably, results were consistent with those of cholesterol synthesis, and BPA exposure upregulated the expression of SREBP‐2 and NPC1L1 in the small intestine, which was markedly reversed by curcumin treatment (Figure 3d–f *p* < .05).

### 3.5 Curcumin inhibited the expression of liver lipogenic genes induced by BPA

**FIGURE 4:**
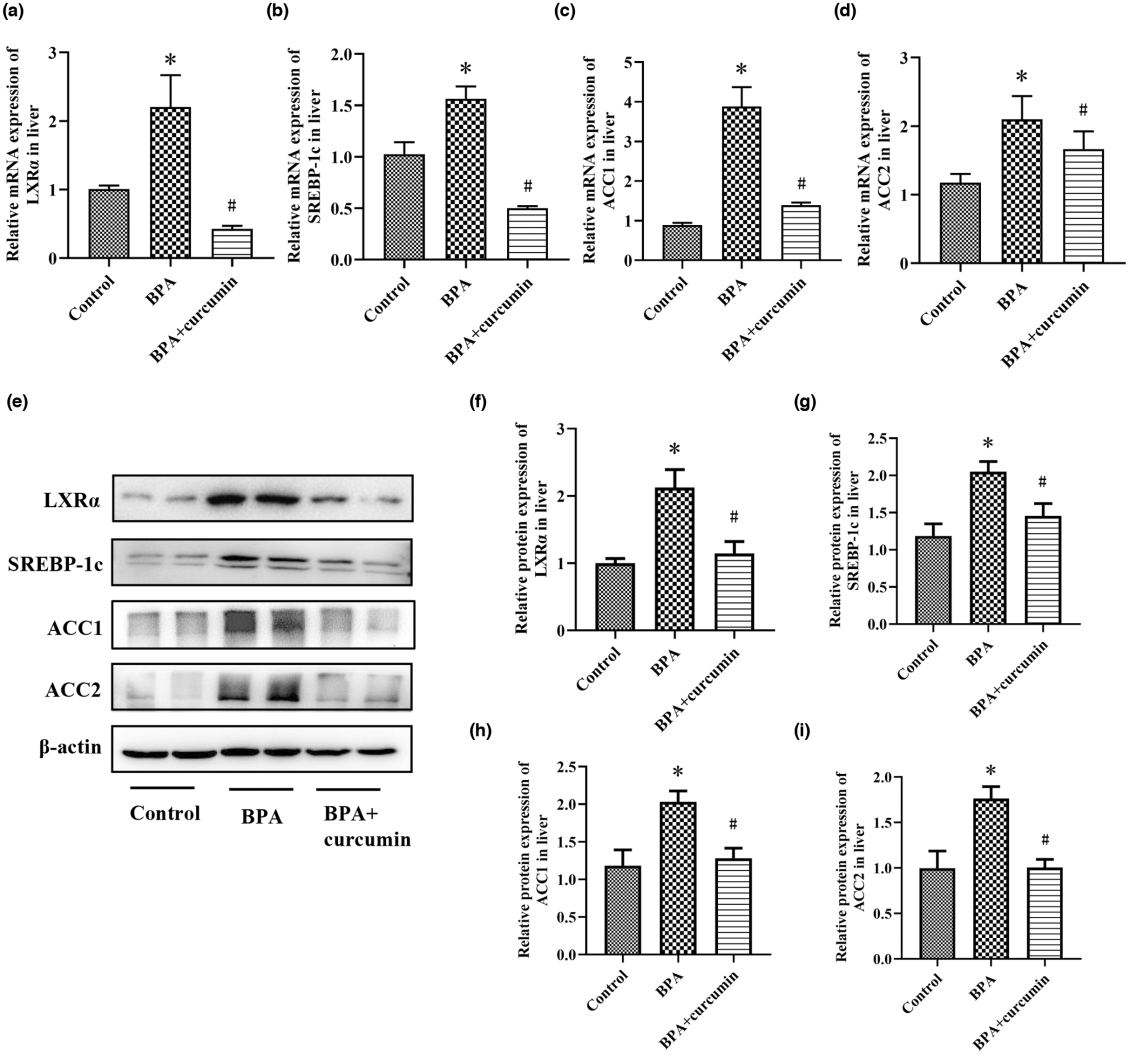
Curcumin inhibited the expression of liver lipogenic genes induced by BPA in CD‐1 mice. Male CD‐1 mice were fed BPA‐contaminated diet with or without curcumin for 24 weeks. (a–d) The mRNA expression levels of LXRα, SREBP‐1c, ACC1, and ACC2 in the liver; (e) The protein expression levels of LXRα, SREBP‐1c, ACC1, and ACC2 in the liver; (f–i) The quantitative analysis of western blotting assay for LXRα, SREBP‐1c, ACC1, and ACC2 expression. Results are expressed as mean ± SEM (*n* = 8), *p < .05 versus control group, and ^#^ *p* < .05 versus BPA group.

Increased cholesterol absorption and synthesis can enhance liver cholesterol accumulation, which further stimulates liver fat synthesis and accumulation, leading to hepatic steatosis (Arguello et al., 2015; Moslehi & Hamidi‐Zad, 2018). Therefore, we further measured the mRNA and protein expression levels of liver lipogenic genes, including LXRα, SREBP‐1c, ACC1, and ACC2 by RT‐qPCR and western blotting assay. Obviously, BPA exposure significantly up‐regulated the mRNA expression of liver LXRα, SREBP‐1c, ACC1, and ACC2 (*p* < .05). It should be noted that curcumin treatment remarkably suppressed the mRNA expression of these lipogenic genes induced by BPA (Figure 4a–d). Western blotting analysis yielded consistent results (Figure 4e–i).

## 4 DISCUSSION

Our current findings demonstrated that curcumin supplementation effectively inhibited BPA‐induced hepatic steatosis by down‐regulating the expression of proteins related to cholesterol absorption and synthesis, which, in turn, reduced liver cholesterol accumulation and alleviated hepatic fat synthesis. This work verified our hypothesis and provided a novel intervention for curcumin as an effective dietary nutrient to prevent BPA‐mediated hepatic steatosis and NAFLD.

As a traditional medicine material and common food spice, curcumin is often used to prevent and treat various diseases. Many studies have demonstrated that curcumin has antioxidant, anti‐inflammatory, and cholesterol‐lowering effects (Feng et al., 2019; Feng, Zou, Zhang, Li, & Lu, 2017; Zhang et al., 2018) and possesses therapeutic potential for metabolic diseases including type 2 diabetes (Thota et al., 2020), cardiovascular diseases (Li et al., 2020), and NAFLD (Khan et al., 2019; Różański et al., 2021). For example, our previous study showed that curcumin reduced serum and hepatic TG and TC levels, and attenuated liver lipid deposition in mice (Feng et al., 2019). We also found that supplementation with curcumin inhibited intestinal cholesterol absorption and lowered serum and liver cholesterol levels in HFD‐fed hamsters and apolipoprotein E knockout mice, preventing HFD‐induced hepatic steatosis and atherosclerosis (Feng, Zou, Zhang, Li, & Lu, 2017; Zhang et al., 2018; Zou et al., 2018). A placebo‐controlled, double‐blind clinical trial showed that curcumin administration improved serum TG and HDL‐C levels, liver transaminases, and steatosis index in overweight subjects with impaired fasting plasma glucose (Cicero et al., 2020). With the deepening of research, studies found that curcumin can resist the damage caused by BPA. Tandon et al. demonstrated that curcumin prevented BPA‐induced neurotoxicity and behavioral deficits in rats via the activation of the Notch signaling pathway (Tandon et al., 2020). Curcumin had also been found to alleviate BPA‐induced insulin resistance in HepG2 cells by inhibiting the JNK/p38 signaling pathway (Geng et al., 2017). Consistent with previous research, our current study revealed that curcumin supplementation substantially attenuated BPA‐induced lipid metabolism disorder and hepatic steatosis in male CD‐1 mice.

The pathogenesis of NAFLD is complicated and has not been completely clarified (Teng et al., 2022). It has been reported that cholesterol metabolism disorder is closely associated with NAFLD (Malhotra et al., 2020). Intestinal cholesterol absorption and hepatic cholesterol synthesis have a crucial role in maintaining body cholesterol homeostasis (Luo et al., 2020). Excessive intestinal cholesterol absorption and hepatic cholesterol synthesis increase serum cholesterol levels and liver cholesterol accumulation, which in turn stimulates liver fat synthesis and promotes hepatic steatosis and NAFLD (Arguello et al., 2015). SREBPs are known as major regulators of adipogenesis and cholesterol metabolism and have important roles in maintaining lipid homeostasis in the body (Park et al., 2018). Among its isoforms, SREBP‐2 is highly expressed in the liver and gut and acts on cholesterol synthesis and absorption. SREBP‐2 mainly regulates the expression of genes related to cholesterol synthesis and absorption, such as HMGCR and NPC1L1 (Howe et al., 2017; Pramfalk et al., 2010). HMGCR is the rate‐limiting enzyme in the cholesterol synthesis pathway, and enhancing the expression of HMGCR can significantly promote cholesterol synthesis, which causes hepatic cholesterol accumulation. SREBP‐2 binds the promoter region of HMGCR to regulate its gene expression, which in turn promotes cholesterol biosynthesis (Howe et al., 2017). In our previous in vitro experiments, we found that exposure to environmentally relevant dose (1 nM ~ 10 nM) of BPA caused a significant increase in SREBP‐2 expression in HepG2 cells, which up‐regulated the expression level of its downstream gene HMGCR, leading to increased cellular cholesterol synthesis (Li, Zhang, Zou, Feng, & Feng, 2019). In addition, we also observed that BPA exposure up‐regulated the expression of SREBP‐2 by down‐regulating its DNA methylation level in C57BL/6 mice, which led to activation of the hepatic cholesterol synthesis gene HMGCR, resulting in increased hepatic cholesterol synthesis and hepatic steatosis (Li, Zhang, Zou, Mai, et al., 2019). Likewise, in the present study, we also observed that exposure to low‐concentration BPA significantly up‐regulated the expression of SREBP‐2 and HMGCR in the liver and induced hepatic steatosis in CD‐1 mice. Moreover, our previous study on Caco‐2 cells models found that exposure to environmentally relevant dose (1 nM ~ 10 nM) of BPA up‐regulated the expression levels of SREBP‐2 and its targeted gene NPC1L1, leading to an increased cholesterol uptake (Feng, Zou, Zhang, Li, Li, & Lu, 2017). NPC1L1 is a critical transporter that mediates intestinal cholesterol absorption (Altmann et al., 2004). Our current experiments also observed that long‐term exposure to low‐dose BPA significantly increased the expression levels of SREBP‐2 and its downstream gene NPC1L1 in the small intestine of CD‐1 mice. The up‐regulation of SREBP‐2 and its downstream genes HMGCR and NPC1L1 by BPA caused increased levels of cholesterol in serum and liver. This, in turn, increased liver cholesterol accumulation. However, supplementation with curcumin markedly suppressed BPA‐induced up‐regulation of SREBP‐2 and HMGCR and NPC1L1 and reduced serum and liver cholesterol concentrations. In line with these researches, we previously showed that curcumin reduced the SREBP‐2 and NPC1L1 expression and cholesterol absorption in the small intestine of HFD‐fed hamsters and apolipoprotein E knockout mice, thereby lowering serum and liver cholesterol levels and preventing HFD‐induced hepatic steatosis and atherosclerosis (Feng et al., 2019; Feng, Zou, Zhang, Li, & Lu, 2017; Zou et al., 2018). It was also found that NPC1L1 expression was dose dependently decreased in Caco‐2 cells after curcumin intervention, along with reduced cholesterol uptake (Feng et al., 2010). Another study on Caco‐2 cells displayed that curcumin directly inhibited intestinal NPC1L1 expression through transcriptionally regulating SREBP‐2 (Kumar et al., 2011). Additionally, curcumin was also shown to suppress clozapine‐induced overexpression of hepatic SREBP‐2 and HMGCR in rats (Liu et al., 2017).

The liver is the central organ of lipid metabolism and hepatic lipid biosynthesis is transcriptionally regulated by LXRα, SREBP‐1c, ACC1, and ACC2. The increased cholesterol absorption and synthesis induced by BPA resulted in liver cholesterol accumulation. It has been reported that liver cholesterol accumulation can cause the activation of hepatic LXRα (Arguello et al., 2015). LXRα is a transcription factor that can stimulate SREBP‐1c expression by binding to SREBP‐1c promoter and lead to liver steatosis (Repa et al., 2000). SREBP‐1c is a key lipogenic transcription factor that activates ACC and is responsible for fatty acid uptake and triglyceride synthesis (Liu et al., 2021). ACC is the first enzyme in the liver de novo lipogenesis (DNL) pathway. There are two isozymes of ACC1 and ACC2 in mammals. Combined inhibition of ACC1 and ACC2 results in DNL reduction, leading to a decrease in liver TG and significant improvement of hepatic steatosis (Kim et al., 2017). The activation of hepatic lipogenic pathway is a critical metabolic change required for hepatic steatosis formation. We also found that dietary BPA exposure induced obvious hepatic steatosis, along with liver cholesterol accumulation and up‐regulation of hepatic LXRα, SREBP‐1c, ACC1, and ACC2. In accordance with our results, Marmugi et al. also demonstrated that exposure to low‐dose BPA induced gene expression related to liver lipid synthesis including LXRα, SREBP‐1c, and ACC1 in adult mice (Marmugi et al., 2012). However, curcumin treatment significantly suppressed the expression of hepatic LXRα, SREBP‐1c, ACC1, and ACC2 and reduced liver fat accumulation induced by BPA.

A limitation of our current study is that we only used male mice. Since gender can have an impact on human NAFLD, our future research should include both male and female mice to obtain results that better reflect the risks of BPA on human health.

## 5 CONCLUSIONS

This study demonstrated the hepatoprotective capacity of curcumin against BPA. Curcumin inhibited BPA‐induced hepatic steatosis by inhibiting intestinal cholesterol absorption and hepatic cholesterol synthesis, then reducing liver cholesterol accumulation, and finally, ameliorating liver lipid biosynthesis and fat accumulation. Our results suggest the application of curcumin as a potential nutrient to protect against BPA‐induced hepatic steatosis. It sheds light on the prevention and treatment of environmental pollutants‐induced metabolic liver diseases by phytochemicals.

## AUTHOR CONTRIBUTIONS

**Ting Hong:** Formal analysis (equal); investigation (equal); methodology (equal); writing – original draft (equal). **Jun Zou:** Conceptualization (equal); funding acquisition (equal); investigation (equal); writing – review and editing (equal). **Jie Yang:** Formal analysis (equal); methodology (equal). **Hao Liu:** Formal analysis (equal); investigation (equal). **Zhuo Cao:** Formal analysis (equal); investigation (equal). **Youming He:** Investigation (equal). **Dan Feng:** Conceptualization (equal); funding acquisition (equal); investigation (equal); methodology (equal); project administration (equal); writing – review and editing (equal).

## FUNDING INFORMATION

This work was supported by grants from the National Natural Science Foundation of China (81973019) and the Natural Science Foundation of Guangdong Province (2022A1515011610, 2020A1515011167, and 2022A1515012268).

## CONFLICT OF INTEREST STATEMENT

The authors declare that they do not have any conflict of interest.

## Supporting information


Appendix S1

## Data Availability

The raw data required to reproduce these findings cannot be shared at this time as the data also form part of an ongoing study.
