# Supplementary material for: Curcumin protects against bisphenol A‐induced hepatic steatosis by inhibiting cholesterol absorption and synthesis in CD‐1 mice
Source: Food Sci Nutr. 2023 Jun 1;11(9):5091–101. doi: 10.1002/fsn3.3468 (PMC10494624; doi:10.1002/fsn3.3468)
Supplement: Supplementary file 1 — Appendix S1 [file FSN3-11-5091-s001.docx]

**1.Supplemental Table**

TABLE S1

Reagents and chemicals involved in the test.

| Chemicals and reagents | Manufacturer |
| --- | --- |
| Anti-SREBP-2  Anti-HMGCR | Abcam, Cambridge, MA, USA  Abcam, Cambridge, MA, USA |
| Anti-NPC1L1 | Santa Cruz Biotechnology, CA, USA |
| Anti-SREBP-1c  Anti-LXRα | Cell Signaling Technology, Danvers, MA, USA  Cell Signaling Technology, Danvers, MA, USA |
| Anti-ACC1  Anti-ACC2 | Santa Cruz Biotechnology, CA, USA  Santa Cruz Biotechnology, CA, USA |
| Anti-β-actin | Abcam, Cambridge, MA, USA |
| BCA protein assay kit | Beyotime, Shanghai, China |
| BPA (≥99% purity) | Sigma, St. Louis. MO, USA |
| Curcumin (≥94% purity) | Sigma, St. Louis. MO, USA |
| Goat anti-rabbit IgG-HRP | Abcam, Cambridge, MA, USA |
| PMSF | Beyotime, Shanghai, China |
| PVDF membrane | Millipore, Boston, MA |
| RIPA | Beyotime, Shanghai, China |
| Sangon Biotech | Sangon Biotech, Shanghai, China |
| Serum ALT Activity Assay | BioSino, Beijing, China |
| Serum AST Activity Assay | BioSino, Beijing, China |
| Serum HDL-C Determination Kit | Applygen Technologies Inc., Beijing, China |
| Serum LDL-C Determination Kit | Applygen Technologies Inc., Beijing, China |
| Serum Total Cholesterol Determination Kit | Applygen Technologies Inc., Beijing, China |
| Serum Total Triglycerides Determination Kit | Applygen Technologies Inc., Beijing, China |
| Tissue Total Cholesterol | Applygen Technologies Inc., Beijing, China |
| Tissue Total Triglycerides | Applygen Technologies Inc., Beijing, China |
|  |  |

**2. Supplemental Method s**

Real-time quantitative polymerase chain reaction (RT-qPCR) assay

Total RNA was extracted from liver tissue using TRIzol reagent (Invitrogen, Carlsbad, CA, USA), and cDNA was synthesised by RNA reverse transcription using the Takara Prime Script™ RT reagent Kit (TakaRa, Otsu, Shiga, Japan). RT-qPCR was performed using Applied Biosystems 7500 System, with a SYBR Green PCR Master Mix kit (TakaRa, Otsu, Shiga, Japan). The necessary primers were synthesised by Sangon Biotech (Sangon Biotech, Shanghai, China). Primer sequences of β-actin, LXRα, SREBP-1C, ACC1 and ACC2 are shown in Table 2. The thermal conditions of PCR were as follows: 1 cycle of 95℃ for 30s, 40 cycles of 95℃ for 5s, 60℃ for 34s, 1 cycle of 95℃ for 15s, 60℃ for 1min, and 95℃ for 15s. The 2−∆∆Ct method was applied to analyse the expression levels of each gene, which was normalised with β-actin.

Western blotting analysis

20 mg of liver tissue was lysed in RIPA (Beyotime, Shanghai, China) containing protease inhibitor phenylmethanesulfonyl fluoride (PMSF) (Beyotime, Shanghai, China) and ground up into homogenate. After centrifugation for 30 minutes (4℃，12000rpm), total protein concentrations in supernatant were measured using BCA protein assay kit (Beyotime, Shanghai, China), then denatured at 100 °C for 6 min. 30 μg protein for each group was loaded to the lane, electrophoretically separated on 10% SDS–PAGE gels at a constant voltage of 100 V for 100 min and transferred to PVDF membrane (Millipore, Boston, MA). The membrane was blocked with 5% BSA for 1.5h at room temperature, followed by incubation with 1:500 diluted primary polyclonal antibodies (anti-β-actin, anti-SREBP-2, anti-HMGCR, anti-NPC1L1, anti-SREBP-1c, anti-LXRα, anti-ACC1, anti-ACC2) for 12h at 4℃. After being washed three times in 30 min, the membranes were incubated with horseradish peroxidise (HRP)-linked goat anti-rabbit IgG secondary antibody (Abcam, Cambridge, MA, USA) at 1:3000 dilution for 2h at room temperature. The specific bands were detected with chemiluminescence (ECL) detection system. Band intensities were quantified by Image J software (NIH, USA). β-actin was used as the loading control.
